# Biological Monitoring and Lung Function Assessment among Workers Exposed to Chromium in the Ceramic Industry

**Published:** 2018-03-19

**Authors:** Fatemeh Kargar Shouroki, Seyed Jamaleddin Shahtaheri, Farideh Golbabaei, Abolfazle Barkhordari, Abbas Rahimi-Froushani

**Affiliations:** ^1^Student Research Committee, School of Health, Shiraz University of Medical Sciences, Shiraz, Iran; ^2^Department of Occupational Health Engineering, School of Health, Tehran University of Medical Sciences, Tehran, Iran; ^3^Institute for Environmental Research, Tehran University of Medical Sciences, Tehran, Iran; ^4^Department of Occupational Health Engineering, School of Health, Yazd University of Medical Sciences, Yazd, Iran; ^5^Department of Epidemiology & Biostatistics, School of Health, Tehran University of Medical Sciences, Tehran, Iran

**Keywords:** Chromium, Exposure, Ceramics, Solid phase extraction

## Abstract

**Background:** Chromium exposure occurs in various industries such as ceramics industry. The main disadvantage of chromium is high toxicity when absorbed by the body. This study aimed to determine and compare urinary chromium values in the pre- and post-shift of ceramic glazers and the control group; investigate urinary chromium value according to the study variables; compare pulmonary function tests between ceramic glazers vs. the control group.

**Study design:** A cross-sectional study.

**Methods:** This study was carried out in Yazd City, central Iran on 49 glazers and 55 office workers in the same workplace as the control group. The urine samples were collected at both start and end of the work shift, and the control group was examined only once. Preparation of samples were conducted by Solid Phase Extraction (SPE). The analyses were performed by inductively coupled plasma-atomic emission spectroscopy (ICP-AES). All the participants filled out a self-administered questionnaire comprising of questions about smoking habits, work shift, skin dermatitis, job title, using mask, ventilation system, duration of exposure, overtime working, age, weight, and height. The lung function tests were performed on study groups.

**Results:** The concentration of chromium after the glazer's shift was 2.73 times higher than the Biological Exposure Index (BEI) recommended by the occupational and environmental health center of Iran. Lung function tests were significantly lower in the glazers compared with the control group (P=0.001).

**Conclusions:** Skin dermatitis and overtime working are the main determinants of high chromium level in glazers.

## Introduction

The raw materials used to produce ceramic are clay and various frits. Frits are glassy materials, insoluble in water, including a combination of mineral materials (feldspar, kaolin, quartz, carbonates and borate oxides), made by melting at high temperature 1500 ºC followed by fast cooling. Frits are crushed to a fine powder and are supplied to the ceramic industry^1^. Raw materials are mixed, dispersed in water then pressed into shape. Next, the products are placed in furnace for water to evaporate and ultimately form ceramic bisque^2^. In the next step, glaze is applied on to the bisque surface.

The major glaze components are silicate network, fluxing composition (alkalis, boron, lead), and various opacifying oxides (zirconium, titanium, etc.), and again baked. The glaze melts during the baking process and seals the ceramic body to reduce porosity, and to produce smooth, waterproof, and shiny surfaces^2^. A wide range of pigments is also used in glazing to produce color. Majority of pigments are inorganic compounds of metal oxides^3^. Metal dust and gas emissions from color pigments can occur during drying, baking, weighing, manufacturing, and handling processes. Trivalent and hexavalent chromium (III .VI) are the most widely used groups of pigments extensively used to produce colors. Chromium oxide (Cr_2_O_3_) is used to produce green color^2^ and chromate VI yellow pigments are responsible for the color of lead chromate^4^.

Being exposed to hexavalent chromium compounds, especially in the chromiumate production and pigment industries can cause asthma and lung cancer^5^ as well as induce leukemia and bone cancer. Skin exposure to hexavalent chromium is known to cause allergic contact dermatitis characterized by skin erythema, pruritus, edema, papule, and scars. Hexavalent chromium compounds are the most potent carcinogenic agents known to human. There is a connection between hexachromium exposure and prostate, stomach, kidney, and bladder and hematopoietic system cancers^6^. Ingesting a high dose of hexavalent chromium can lead to renal failure, characterized by proteinuria, anuria, and hematuria. In addition to workers in this industry, the use of ceramic crockery is one of the major routes of being exposed to toxic metal in the general population^2^. Some studies have shown higher concentration of chromium in pigment workers compared to controls^7,8^. For instance, in McAughey's study in pigment workers in England, that exposed to strontium and lead chromate, the concentrations of chromium in the urine ranged from 1.8 to 575 μg chromium/g creatinine in comparison to a level of <0.5 μg chromium/g creatinine for control group^8^.

In this study, solid phase extraction (SPE) technique was used to prepare urine samples. This method was optimized for chromium and other metals in our previous studies^9-11^. Exposure to chromium increases the prevalence of respiratory symptoms and decrease lungs’ functional capacities^4^. However, to date little attention has been paid to exposure to chromium in the ceramic industry and there is special interest in silica exposure assessment in Iran.

The present study aimed to determine the urinary chromium values in the pre-post-shift of the glazers in comparison with the control group and to investigate urinary chromium value according to the study variables. Besides, we compared the pulmonary functions test between the glazers and the control groups.

## Methods

This cross-sectional study was conducted in Yazd City, central Iran among 49 subjects with a mean age of 30.67 yr (range 22 to 50 yr), working in 15 tile factories and 6 pottery workshops as glazers. Total of 55 office workers in the same workplaces not exposed to chromium were chosen as controls with a mean age of 32.61 yr (range 23 to 50 yr). The sample size was determined based on a previous study^12^. None of subjects had any occupational or non-occupational exposure to dust or other substances that could lead to pulmonary disorders. In order to control the confounding factors, subjects with chronic respiratory diseases, asthma, lung infections were excluded from the study.

The work shift duration was 8h with two shifts per day and all the participants were men. Prior to data collection, the participants were informed about the aim of this study and had signed a written informed consent.

The questionnaire contained questions on smoking habits, morning/evening shift, skin dermatitis, job title, using mask, ventilation system, duration of exposure (years working as glazer), overtime working, age, weight, and height. Smoking variable was eliminated since none of the subjects were smokers.

To estimate chromium concentration in our subjects, two urine samples were taken, one at the beginning (pre-shift), and the second sample at the end of their work shift (post-shift), total of 98 samples. From the control group, urine sample was only collected once (55 samples). Overall, 20 ml urine sample was collected in sterile containers to avoid contamination, and stored in a fridge to be analyzed a week later.

Chromium in biological samples can be extracted in a variety of ways. Sample preparation techniques such as liquid-liquid extraction (LLE), Soxhlet extraction and supercritical fluid extraction (SFE) are time-consuming and error-prone aspects prior to analysis^10^. Therefore, more precise and sensitive methods are required to measure heavy metals in biological samples. In contrast, solid phase extraction methods using silica has proven useful in simplifying sample preparation prior to analytical technique^9^. SPE using mini columns filled with resin was optimized regarding sample pH, loading flow rate, ligand concentration, sample volume, elution solvent, elution volume, amount of resins, and sample matrix interferences in our previous study^9-11^.

### Sample preparation

SPE was conducted by packing the cartridges with 500 mg amberlite XAD-7 resin. The columns were washed twice with ethanol, water, and 1 M HCl, adjusted to pH 9; urine samples were chelated with ammonium pyrrolidine dithiocarbamate (APDC). Then, the samples were diluted to 25 ml with distilled water and passed through the resin at a flow rate of 5 ml/min under gravity force. The column was then washed out with water and 1 M NaOH. The retained analyte was eluted with 15 ml of 2 M HNO_3_. The solution was then analyzed for chromium, using the inductively coupled plasma-atomic emission spectroscopy (ICP-AES) (SPECTRO, ARCOS, and Germany).

### Pulmonary function tests

Spirometry was conducted between the glazers and the control group with a calibrated portable vitalograph spirometer (model 2120). For the purpose of spirometry, the subjects were asked to stand on their feet. In this test, forced vital capacity (FVC), forced expiratory volume 1 sec (FEV1), forced expiratory flow between 25%-75% of the FVC (FEF _25-75_), and the ratio of FEV1/FVC as parameters of the breathing capacity of the lungs were determined. To increase the reliability of the measurements, each subject was tested twice with a 15-min break in between, and the highest number was recorded.

### Statistical analysis

Data management and analysis *were performed*  using SPSS 16.0 (Chicago, IL, USA). In order to estimate the differences between the glazers and the control group, *t* -test was used. Changes in pre-post shift values were compared using paired *t* -tests. To study the effect of each variable on post-shift value after adjusting the pre-shift, covariance (ANCOVA) was used. *P* -value of less than 0.05 was considered significant.

## Results

Occupational exposure to chromium was determined by biological monitoring in 49 glazers. Demographic data and the results of lung function tests of all the participants are listed in Table 1. The two studied groups had similar demographic characteristics. All values of spirometric parameters in the glazers were significantly lower than those of the control group (Table 1).

**Table 1:** Comparison of demographic characteristics and respiratory parameters of the studied groups

**Variables**	**Glazers**		**Control**		***P*** **value**
	**Mean**	**SD**	**Mean**	**SD**	
Age (yr)	30.67	5.88	32.61	7.13	0.124
Height (cm)	174.53	8.63	176.42	9.06	0.263
Weight (kg)	77.44	11.72	78.25	10.64	0.704
Body mass index (kg/m^2^)	25.59	4.39	25.33	4.29	0.762
Overtime (hour)	10.05	14.92	0.86	2.74	0.001
Duration of exposure (month)	51.53	33.44	76.32	67.92	0.021
FVC (L)	4.24	0.67	4.74	0.66	0.001
FEV1(L)	3.52	0.71	4.25	0.74	0.001
FEV1/FVC (%)	80.99	8.16	88.85	5.61	0.001
EF 25-75 (L)	3.47	0.83	4.49	0.69	0.001

Table 2 presents the mean chromium concentration (µg/L) in the glazers and the control group urine samples. Mean concentrations of chromium in the urine sample of the participants at the beginning and at the end of their shift was 44.18 μg/l (range=28-127 μg/l), and 68.32 μg/l. (range=29-405 µg/L), respectively. This difference was statistically significant (*P* =0.022).

**Table 2:** Comparison between the means of urinary chromium pre-and post-shift samples in the glazers and the control group

**Control (1)**		**Pre-shift glazer (2)**		**Post-shift glazer (3)**		***t*** **-test,** ***P*** **-value**		
**Mean**	**SD**	**Mean**	**SD**	**Mean**	**SD**	**(1) vs (2)**	**(1) vs (3)**	**(2) vs (3)**
13.51	8.67	44.18	23.38	68.32	73.44	0.008	0.001	0.022

The concentration of chromium was significantly higher in the pre-post shift of the exposed group in comparison to the control group (44.18 ±23.38 and 68.32 ±73.44 versus 13.51 ±8.67) (*P* =0.008) (*P* =0.001).

The concentration of urinary chromium in pre-post shift of glazers according to the variables are reported in Table 3. In glazers who worked in the evening shift, higher urine chromium was found in comparison to those who worked in the morning shift (86.64 µg/L vs. 50.02 µg/L). ANCOVA showed that, after adjusting for baseline value, no significant differences in urinary chromium levels were found between different shifts (Table 4).

**Table 3:** Mean and standard deviation (SD) of urinary chromium measured in pre- and post-shift of the glazers

**Factor**	**Number**	**Pre-Shift**		**Post-Shift**	
		**Mean**	**SD**	**Mean**	**SD**
Work shift					
Morning	25	42.84	21.59	50.02	36.34
Evening	24	45.58	25.52	86.64	94.77
Skin dermatitis					
No	33	43.75	24.79	53.03	60.73
Yes	16	45.06	20.93	139.83	90.75
Job title					
Pottery glazer	14	52.86	33.84	56.93	31.54
Tile glazer	35	40.71	17.04	72.71	84.27
Using Mask					
No	20	48.25	28.84	76.59	67.58
Yes	29	41.38	18.77	64.09	76.92
Ventilation system					
Not standard	37	43.43	22.33	79.88	85.23
Standard	12	46.51	27.43	41.33	9.98
Duration of exposure (month)				
≤24	29	42.93	26.09	38.31	6.23
25-60	11	43.12	14.09	65.67	55.74
≥61	9	46.56	30.23	101.31	109.15
Overtime working (hour)				
No	29	49.32	29.52	49.72	35.74
1-20	11	38.36	8.38	59.91	52.33
21-60	9	36.22	5.51	116.44	133.11

**Table 4:** Results of ANOCOVA for post-shift urine chromium by independent factors controlled for pre-shift chromium

**Source**	**F**	***P*** **value**
Work shift	3.62	0.062
Skin dermatitis	6.06	0.023
Job title	0.31	0.642
Using mask	0.17	0.684
Ventilation system	2.33	0.132
Duration of exposure (month)	3.01	0.063
Overtime working (hour)	5.06	0.014

The urinary concentrations of chromium were higher in glazers with skin dermatitis compared to the glazers without skin dermatitis (139.83 µg/L vs. 53.03 µg/L) (Table 3). After adjusting pre-shift chromium, a significant relationship was found between exposure to chromium and skin dermatitis (*P* =0.023) (Table 4).

The tile glazers group had more urinary chromium than the pottery glazers group (72.71 µg/L vs. 56.93 µg/L). After adjusting for pre-shift chromium, the effect of job title on post-shift chromium was not significant.

Glazing in a workplace with standard ventilation system and using protective masks reduces the urinary chromium concentration in compare with glazing without protection and standard ventilation system (41.33 µg/L vs. 79.88 µg/L and 64.09 µg/L vs. 76.59 µg/L, respectively). However, after adjusting for pre-shift chromium, the effect of mask and ventilation on the post-shift chromium was not significant (Table 4).

The duration of exposure effect on the urinary level of chromium showed that the glazers with prolonged exposure (>61 months) had a higher urine chromium level in comparison with those exposed to chromium less than 24 months (101.31 µg/L vs. 38.31 µg/L) (Table 3). After adjusting for pre-shift chromium, the effect of exposure duration on post-shift chromium was not significant (Table 4). Chromium urinary concentration in the glazers appeared to be higher when they worked overtime (more than 20 h a month) (116.44 µg/L vs. 49.72 µg/L), and the effect of overtime working on post-shift chromium was significant (*P* =0.014) (Table 4).

## Discussion

Chromium glazes are used in ceramic industrial. The main disadvantage to the use of chromium is its high toxicity into the body. Therefore, assessment of glazers’ exposure to chromium has been important in toxicology. Optimized preparation method was validated with different spiked urine samples and the obtained recoveries of metal ions were greater than 92% ^9-11^.

Our study revealed that chromium values in the urine had significantly increased after a working shift in comparison to their pre-shift values (*P* =0.022), and exposure to chromium in both per-post shifts of the glazers was significantly higher than the control group as well as the Biological Exposure Index (BEI) set by the occupational and environmental health center of Iran (25 µg/l)^13^.

Our results were in consent with study that reported chromium concentration was low in the ceramic factories air, however, the upper confidence limit (CLU) in the job categories involved in milling and mixing of pigment powder was higher than the threshold limit value (TLV)^14^.

Urinary excretion of chromium from 101-738 µg/1 has been reported in pigment industry workers due to exposure to chromium salt and most of the workers complained of dermal itching^7^.

Our results showed that the highest values of excreted chromium in the urine were among the subjects with skin dermatitis. Hand dermatitis was observed among enamellers and decorators in the ceramics industry^15^. In fact, a number of studies have found substantial evidence indicating a positive relationship between exposure to chemicals used in glazing and contact dermatitis^16,17^.

No significant differences were found between pottery and tile glazers and between morning and evening shifts. The effect of using mask and ventilation system on the urinary chromium content was also evaluated. Not using mask or respirators and working in confined space with inadequate ventilation system was associated with higher levels of chromium. Several modifications should be made to ventilation systems and training on the use of personal protective equipment should be provided.

High values of chromium were observed in glazers with prolonged exposures. A possible explanation for lack of significant results may be due to the small sample size; however, this was due to limited number of glazers. In the present study, the higher values of chromium were recorded among glazers with more overtime working hours.

The other aim of this study was to investigate the effects of chromium on lung function tests. Glazers in comparison to the control group showed significant decrease in all parameters of pulmonary function, particularly in FEV1 and the ratio of FEV1/FVC. Occupational exposure to chromium dust or fume may induce chronic obstructive pulmonary disease (COPD). Hence, there are several possible explanations for this result such as the effect of other substances in the glaze, and the raw material used like silica.

For instance, significant increases were reported in the COPD in ceramic industry workers^18^. A similar study performed on 33 male workers exposed to raw materials in ceramic production with exposure duration of 6.5 yr, reported a significant decrease in some parameters of spirometry that could be due to silica contents in the ceramic^19^.

Chromium can be released from ceramic ware in contact with hot food or acidic food^2-2,2^.

Recent developments in pigment production have heightened the need for reduction of toxic pigments. For example, in order to reduce toxicity of black pigments containing chromium/iron/nickel, use of Mg and Zn instead of nickel and low content of Cr in black pigments was proposed^23^.

## Conclusions

Findings indicate high levels of chromium in the ceramic industrial. Since none of the subjects were smokers, cigarette smoking cannot be the cause of differences in the spirometry results between the two groups. Hence, changes in pulmonary function parameters can be attributed to the exposure to chromium in glazers. Engineering controls and use of personal protective equipment is needed to reduce workers’ exposure to chromium.

## Acknowledgements

The authors wish to thank the Research Consolation Center (RCC) at Shiraz University of Medical Sciences for their invaluable assistance in editing this manuscript.

## Conflict of interest statement

The authors declare that there is no conflict of interest.

## Funding

This research has been supported by Tehran University of Medical Sciences and Health Services grant (project no. 11/2/88/8911). The authors acknowledge the university supports.

## Highlights

- The chromium level in urine of glazers was significantly higher than that of control individuals.
- The mean values of parameters of pulmonary in the glazers were significantly lower than those of control individuals.
- Skin dermatitis and overtime working were the main determinants of high chromium level in glazers.
